# Sleep disorders predict the 1-year onset, persistence, but not remission of psychotic experiences in preadolescence: a longitudinal analysis of the ABCD cohort data

**DOI:** 10.1007/s00787-022-01966-z

**Published:** 2022-03-16

**Authors:** Sarah Reeve, Vaughan Bell

**Affiliations:** 1 Norwich Medical School, University of East Anglia, Norwich, UK; 2 Department of Clinical, Educational, and Health Psychology, University College London, London, UK; 3 South London and Maudsley NHS Foundation Trust, London, UK

**Keywords:** Sleep, Psychotic experiences, Preadolescence, Longitudinal, Stimulant medication

## Abstract

The relationship between sleep disorder and psychotic experiences in preadolescence has not been extensively studied despite the potential for intervention. The current study addressed this relationship using the Adolescent Brain and Cognitive Development (ABCD) cohort, which provided baseline data from 11,830 10- to 11-year-old; for 4910 of these, 1-year follow-up data were also available. A set of pre-registered multi-level regression models were applied to test whether (a) sleep disorder is associated with psychotic experiences at baseline; (b) baseline sleep disorder predicts psychotic experiences at follow-up; (c) the persistence of sleep disorder predicts persistence of psychotic experiences at follow-up; d) the remission of sleep disorder predicts the remission of psychotic experiences at follow-up. After controlling for potential confounders, sleep disorder was associated with psychotic experiences cross-sectionally (OR = 1.40, 95% CI 1.20–1.63), at 1-year follow-up (OR = 1.32, 95% CI 1.11–1.57), and the persistence of sleep disorder predicted the persistence of psychotic experiences (OR = 1.72, 95% CI 1.44–2.04). However, remission of sleep problems did not predict remission of psychotic experiences (OR = 1.041, 95% CI 0.80–1.35). The results indicate that sleep disorders in preadolescence are common and associated with psychotic experiences, although the lack of co-remission raises questions about the mechanism of association. However, given these findings, and existing evidence in later adolescence and adults, further investigation of sleep as a preventative mental health intervention target in this age group is warranted.

**Supplementary Information:**

The online version contains supplementary material available at 10.1007/s00787-022-01966-z.

## Introduction

Sleep disorders are hypothesised to be a significant causal factor in the development and maintenance of psychosis [1, 2]. Experimental research in adults supports that sleep disruption increases psychotic experiences [3], and that treating sleep disorders reduces psychotic experiences in adults [4]. While the relationship between sleep and psychotic symptoms is thought to be bidirectional, longitudinal studies in adults have indicated that sleep disorders predict later psychotic symptoms to a greater extent than vice versa [5, 6]. These and other findings have raised the possibility of targeting sleep disorders as a preventative intervention in mental health [4, 7, 8].

It is known that psychotic-like experiences are roughly 10 × more common in children than they are in adults (reported by 66% of 7- to 8-year old children compared to 5.8–7.2% of adults; [9–11]). Not all childhood psychotic experiences are pathological [12]; nevertheless, persistence, distress, and amount of different psychotic experiences reported in childhood are associated with higher risk of developing adult psychotic disorder [13, 14] and a predictor of poor physical and mental health more generally later in life [15–17]. The age at which psychotic experiences are reported is also positively related to risk of later psychotic disorder. Reporting psychotic experiences at age 10–11years is associated with a 5 × increased likelihood of developing a later psychotic disorder [14], whereas reporting these experiences at ages 14–17 years is associated with a 10 × increased likelihood [13]. This set of findings has been encompassed under a ‘proneness to persistence’ model of psychosis, with these non-clinical psychotic experiences existing on a continuum with psychotic disorders [10].

The relationship between sleep disorders and psychotic experiences in children is less well researched than in adults [1]. However, many studies have demonstrated that children or adolescents with sleep disturbances are more likely to report psychotic experiences [18–20]. Several large population cohort studies have reported that sleep problems during childhood predict psychotic experiences contemporaneously and across adolescence [19, 21–23]. Furthermore, as in adults, sleep problems in children are linked to a wide range of other negative mental and physical health outcomes [24–26].

Despite this evidence indicating that sleep disorders have an important role in the onset of psychotic experiences during childhood and adolescence, it is less clear whether sleep disorders contribute to persistence of psychotic experiences in this age group. This is particularly important given that persistence is a particularly strong predictor of poor outcome. It is also notable that a high proportion of both sleep problems and psychotic experiences remit during adolescence [27]—yet as with persistence, whether sleep problems have an influence on remission of psychotic experiences is unknown. The large cohort studies listed above have rarely measured both sleep and psychotic experiences at multiple time points (with sleep usually assessed only at younger ages and psychotic experiences usually assessed only in teenage years) making it difficult to explore their concurrent relationship.

## The current study

We used the Adolescent Brain and Cognitive Development study cohort data (release 2.0.1) to investigate the relationship between sleep and psychotic experiences in a cohort of 9- to 11-year-old to address a set of pre-registered hypotheses:

1. Do sleep disorders and psychotic experiences co-occur in 9- to 10-year-old children?
2. Do sleep disorders predict later onset of psychotic experiences?
3. Do sleep disorders predict persistence of psychotic experiences?
4. Does remission of sleep disorders predict remission of psychotic experiences?

These were examined use a pre-registered set of linear and logistic regression models applied to the baseline and 1-year follow-up data using a pre-registered analysis plan (https://osf.io/8ks72/).

## Method

### Recruitment

The Adolescent Brain and Cognitive Development (ABCD; https://abcdstudy.org/) 2.0.1 release study data were used in the current analysis [28, 29]. The ABCD study is an ongoing longitudinal cohort study aimed at recruiting a representative sample of US children. At the initial stage, all families of children aged 9–10 years in geographic catchment area of study sites across the USA were contacted via schools with information about the study. Volunteer families were then screened for inclusion. Participants were purposively recruited to match national US sociodemographic factors, for example utilising targets for each of five major race/ethnicity classifications (white, African-American, Hispanic, Asian, all other). The 2.0.1 release includes baseline data on 11,873 individuals, and 1-year follow-up from 4951 of those participants, with follow-up data from the remainder of the cohort to be released at a future date. Full details of the ABCD study design are available in a special issue of Developmental Cognitive Neuroscience [28].

### Measures

#### Psychotic experiences

Participants’ psychotic experiences were assessed using the Prodromal Questionnaire-Brief Child version (PQ-BC). This measure was validated within the current ABCD dataset showing high reliability (Cronbach’s alpha = 0.863 for total score and 0.873 for distress subscale; [30]. The PQ-BC is a self-report questionnaire assessing presence and distress associated with psychotic-like symptoms in children. For each psychotic experience the child is asked if they experience it (yes/no), and if they do, how much it distresses/bothers them on a pictographic 1 to 5 scale showing a human cartoon figure in various levels of distress. This questionnaire yields two outcome variables—the sum of symptoms endorsed (range 0–21) and the sum of distress reported for those symptoms (range 0–126).

For the current study, both continuous and dichotomous outcomes of the sum of symptoms were used. For the dichotomous outcome, the symptom score was transformed to a categorical 0–1 variable, where 1 indicates the presence of at least one psychotic symptom and 0 indicates no psychotic symptoms present. When applied to baseline and follow-up, this was then used to derive variables indicating ‘new onset’ of psychotic symptoms (i.e. 0 at baseline, 1 at follow-up) ‘persistence’ of psychotic symptoms (i.e. 1 at both time points), and ‘remission’ (1 at baseline, 0 at follow-up).

As an addition to our analysis plan (see Alterations to the pre-registered statistical plan for further details and rationale), we derived a count of *distressing* psychotic experiences which was dichotomised to represent the presence/absence of at least one distressing psychotic symptom (≥ 2 score on distress rating).

#### Sleep disorders

Presence of sleep disorders was assessed using the Sleep Disorder Scale for Children (SDSC), which is a parent-reported questionnaire assessing the presence of a range of sleep disorder symptoms in children [31]. It is composed of 26 Likert items assessing the frequency of various disturbances over the past 6 months on a 1 to 5 Likert scale (1 = never, 5 = always/daily experiencing a particular issue). The total score provides a measure of sleep disturbance, for which a cutoff point at 39 has sensitivity of 0.89 and specificity of 0.74, correctly identifying 73.4% of a control group and 89.1% of sleep disordered participants.

This cutoff was used to categorise participants according to absence or presence of disturbed sleep. The categorical score at baseline and follow-up was then used to derive variables indicating ‘persistence’ of sleep disturbance (i.e. present at both time points), ‘remission’ (present at baseline, absent at follow-up), and ‘onset’ (absent at baseline, present at follow-up).

#### Potential confounders: sociodemographic, IQ, and medication variables

Further variables were used from the ABCD dataset to index potential confounders, defined as factors that can independently influence each of the variables of interest (sleep and psychotic experiences).

- Male gender and non-white ethnicity are associated with a higher likelihood of reporting both sleep problems [32] and psychotic experiences [33]. Gender (Male/Female), ethnicity (white/black/Hispanic/Asian/other) were reported within the basic demographic questionnaires of the study. Ethnicity was re-coded into white/non-white for the purposes of all analyses.
- Lower socioeconomic status is also associated with increased likelihood of psychiatric disorder [34] and shorter sleep duration [35]. Socioeconomic status was indexed by using the sum score (range = 0–7) of seven yes/no items in the parent demographic survey questions relating to experiences of family hardship (e.g. “*in the past 12 months has there been a time when you and your immediate family needed food but couldn't afford to buy it or couldn't afford to go out to get it?*”), with higher scores on this sum scale indicating lower socioeconomic status. Neighbourhood deprivation was also assessed using the area deprivation index of the home address, which provides a national percentile value (range 1–100) with higher values indicating higher levels of deprivation.
- Family conflict is also associated with sleep problems [36] and psychotic experiences [37]. Family conflict was indexed by the nine-item family conflict subscale of the family experiences. Each item is reported by parents as true or false (e.g. *“We fight a lot in our family”*), with higher values indicating higher levels of conflict (range = 0–9).
- Lower IQ scores and prescription of stimulant medications have been reported to have associations with psychotic experiences [38] and, especially for stimulant medications, with sleep problems [39]. Child IQ was assessed using the WISC-V matrix reasoning subscale score (range = 1–19), with higher values indicating higher IQ. Medication fields were searched for any stimulant medications (e.g. *“Methylphenidate”*) and their trade names (e.g. *“Ritalin”*) with absence or presence coded as a dichotomous 0/1 variable.

Notably, depression and anxiety were not included as potential confounders as these are consistently found to act as mediators in the causal pathway between sleep and psychotic experiences (e.g. [3]). Therefore, if included in statistical models as confounders, this would likely result in an underestimate of the relationship between sleep and psychotic experiences which was the primary focus of the current investigation.

### Analysis

The pre-registration document and the analysis code used in this study are available online at the following link: https://osf.io/8ks72/. R version 3.6.2 was used for all analyses. A list of packages and version numbers used can be found in Supplementary Material 1. The pre-registration was completed before the ABCD 2.0 data release, i.e. before the 1-year follow-up data was made available.

For each research question a set of planned regression analyses were pre-specified. In each case, the regression model was first estimated with only the key explanatory (sleep) and dependent (psychosis) variables. If a significant association was found, the analysis was repeated with potential confounders added (simultaneously) to test the robustness of the hypothesised association.

The following four research questions were tested:

1. Do sleep disorders and psychotic experiences co-occur?

This was investigated cross-sectionally within baseline using (a) a linear regression to test continuous association between sleep symptoms and psychotic experiences (b) a logistic regression to test if sleep symptoms (continuous) predicted presence of psychotic experiences (dichotomous) and (c) a logistic regression to test if presence of sleep disorder (dichotomous) predicted presence of psychotic experiences (dichotomous).

2. **Do sleep disorders predict later psychotic experiences?**

This was investigated by logistic regression models testing if the presence of sleep disorder (a) at baseline and (b) at both baseline and follow-up predicted psychotic experiences at 1-year follow-up.

3. **Do sleep disorders predict **persistence** of psychotic experiences?**

This was investigated by logistic regression models testing if the presence or persistence of sleep disorder predicted persistence (i.e. presence at both baseline and follow-up) of psychotic experiences.

4. **Does remission of sleep disorders predict remission of psychotic experiences?**

This was investigated by logistic regression models testing if the remission of sleep disorders (i.e. present at baseline, absent at follow-up) also predicted remission of psychotic experiences using logistic regression.

#### Unregistered analyses

To additionally examine potential associations with distressing psychotic experiences, we completed the planned regression analyses with an alternative outcome: the presence/absence of at least one *distressing* psychotic experience. This followed further published analyses of the PQ-BC responses in the ABCD dataset that advised inclusion of distress to reduce false positives and thereby increase validity of the measure [30, 40].

In addition, we altered our analysis plan across all regression analyses based on ABCD analysis guidance by (i) including clustering by site and family to create multi-level models (otherwise known as mixed-effect models) that included site and family as random intercept factors [41], and (ii) repeating the analyses with scaling by population weights within the analyses (reported in supplementary materials).

## Results

### Demographic and descriptive results

Table 1 displays the descriptive statistics for the study. Most descriptive variables remained relatively consistent between baseline and follow-up; however, the follow-up group had a preponderance of white participants (59.5% compared to 52.1%) and fewer black participants (9.3% at follow-up compared to 15.0% at baseline).

**Table 1:** Demographic and descriptive variables

Descriptive statistics	Baseline, *n* = 11,830	12-month follow-up, *n* = 4910
Age—mean (SD)	9y10m (7.4 m)	11y0m (7.6 m)
Gender—*n* male (%)	6162 (52.1%)	2565 (52.2%)
Ethnicity—*n* (%)		
White	6161 (52.1%)	2923 (59.5%)
Black	1769 (15.0%)	457 (9.3%)
Hispanic	2391 (20.2%)	930 (18.9%)
Asian	250 (2.1%)	115 (2.3%)
Other	1238 (10.5%)	485 (9.9%)
Socioeconomic status scale—mean (SD)	0.47 (1.1)	0.38 (1.0)
Child IQ Scaled Score—mean (SD)	9.86 (3.0)	10.14(2.9)
Neighbourhood deprivation percentile—mean (SD)	39.22 (27.3)	36.23 (25.3)
Family conflict scale—mean (SD)	2.54 (2.0)	2.47 (1.9)
Stimulant medication prescribed—*n* (%)	722 (6.1%)	322 (5.6%)
PQ-BC total—mean (SD)	2.63 (3.6)	1.71 (3.0)
PQ-BC distress—mean (SD)	6.31 (10.6)	4.05 (8.7)
SDSC total—mean (SD)	36.53 (8.2)	36.35 (7.9)
Derived variable counts		
Sleep disorders present (≥ 39 cutoff on SDSC)—*n* (%)	3602 (30.4%)	1392 (28.4%)
Sleep disorders onset (absent at baseline, present at follow-up)—*n* (%)		482 (9.8%)
Sleep disorders persist (present at both baseline and follow-up)—*n* (%)		909 (18.5%)
Sleep disorders remit (present at baseline, absent at follow-up)—*n* (%)		490 (10.0%)
Psychotic experiences present (≥ 1 on PQ-BC total)—*n* (%)	7278 (61.5%)	2298 (46.8%)
Psychotic experiences onset—*n* (%)		473 (9.6%)
Psychotic experiences persist—*n* (%)		1808 (36.8%)
Psychotic experiences remit—*n* (%)		1208 (24.6%)

*SDSC* Sleep Disorder Scale for Children, *PQ-BC* Prodromal Questionnaire-Brief Child version, *SD* standard deviation

Sleep disorders were present in the cohort at similar proportions at baseline and follow-up (30.4% and 28.4%, respectively). However, this masks some turnover, with roughly a third of the sleep disorder cases at follow-up representing ‘new onset’ cases (9.8%) to replace a similar proportion that remitted over the year (10.0%). For psychotic experiences there is a clear decline (61.5% of children reporting at baseline, versus 46.8% at follow-up), a result of the high remittance rate (24.6%) compared to new onset (9.6%). This is also reflected in the reduced average PQ-BC score at follow-up versus baseline.

### RQ1: Do sleep disorders and psychotic experiences co-occur in childhood?

The regression results associated with this research question can be found in Table 2. They illustrate that sleep disorders and psychotic experiences are significantly and strongly associated within the baseline time point. Sleep disorder symptoms above cutoff is associated with an OR of 1.50 (95% CI 1.30–1.74) of at least one psychotic experience being present. The OR remains significant but reduces slightly (to 1.40, 95% CI 1.20–1.63) once the control variables are added (RQ1f). Results were similar in the additional analyses using presence of at least one distressing psychotic experience as the outcome in the unadjusted analysis (OR 1.49; 95% CI 1.29–1.71) and in the adjusted analysis (OR 1.39, 95% CI 1.20–1.60). Tables with the full details of all analyses related to at least one distressing psychotic experiences are show in Supplementary Table 2.

**Table 2:** Regression analyses of co-occurrence of sleep disorders and psychotic experiences at baseline (T0)

Model (outcome)	Parameters	*B*	95% CI	*p* value	AIC
Model 1a (PQ-BC total T0) ^a^	SDSC total T0	0.048^a^	0.04, 0.06	< 0.001	25.850

Model (outcome)	Parameters	Odds	95% CI	*p* value	AIC
Model 1b (PQ-BC cutoff T0) ^b^	SDSC total T0	1.028	1.02, 1.04	< 0.001	6320
Model 1c (PQ-BC cutoff T0) ^b^	SDSC cutoff T0	1.503	1.30, 1.74	< 0.001	6330

Model (outcome)	Parameters	B	95% CI	*p* value	AIC
Model 1d (PQ-BC total T0) ^a^	SDSC total T0	0.037	0.02, 0.05	< 0.001	24,131
	Gender_(RV=Male)_	–0.289	− 0.49, − 0.09	0.004	
	Ethnicity_(RV=White)_	0.039	− 0.04, 0.12	0.347	
	Socioeconomic status	0.183	0.08, 0.29	0.001	
	Neighbourhood deprivation	0.011	0.01, 0.02	< 0.001	
	IQ	–0.062	− 0.10, − 0.03	< 0.001	
	Family conflict	–0.003	− 0.06, 0.05	0.927	
	Stimulant medication	0.603	0.21, 1.00	0.003	

Model (outcome)	Parameters	Odds	95% CI	*p* value	AIC
Model 1e (PQ-BC cutoff T0) ^b^	SDSC total T0	1.022	1.01, 1.03	< 0.001	5860
	Gender_(RV=Male)_	0.829	0.73, 0.94	0.005	
	Ethnicity_(RV=White)_	1.003	0.95, 1.06	0.897	
	Socioeconomic status	1.124	1.04, 1.21	0.003	
	Neighbourhood deprivation	1.009	1.01, 1.01	< 0.001	
	IQ	0.937	0.92, 0.96	< 0.001	
	Family conflict	0.998	0.96, 1.03	0.913	
	Stimulant medication	1.247	0.94, 1.65	0.121	
Model 1f (PQ-BC cutoff T0) ^b^	SDSC cutoff T0	1.401	1.20, 1.63	< 0.001	5863
	Gender_(RV=Male)_	0.828	0.73, 0.94	0.004	
	Ethnicity_(RV=White)_	1.006	0.95, 1.06	0.826	
	Socioeconomic status	1.131	1.05, 1.22	0.001	
	Neighbourhood deprivation	1.009	1.01, 1.01	< 0.001	
	IQ	0.937	0.92, 0.96	< 0.001	
	Family conflict	1.003	0.97, 1.04	0.890	
	Stimulant medication	1.264	0.96, 1.67	0.098	

*SDSC* Sleep Disorder Scale for Children, *PQ-BC* Prodromal Questionnaire-Brief Child version, *B* standardised beta, *CI* confidence intervals, *AIC* Akaike information criterion, ^a^linear regression, ^b^logistic regression

### RQ2: Do sleep disorders predict onset of psychotic experiences?

The results of the logistic regression analyses on this research question are shown in Table 3. Sleep disorder symptoms at baseline were found to predict psychotic experiences 1-year later (OR 1.62, 95% CI 1.41–1.86), even once sleep disorder symptoms at the later time point were controlled for (OR 1.41 95% CI 1.20–1.65). This association remained after potential confounding variables were added (OR 1.32, 95% CI 1.11–1.57). Again, results were similar when presence of distressing psychotic experiences was used as the outcome in both the unadjusted (OR 1.39, 95% CI 1.18–1.64) and adjusted analysis (OR 1.33, 95% CI 1.12–1.58).

**Table 3:** Regression analyses of whether sleep disorders at baseline predict onset of psychotic experiences at 12 months

Model (outcome)	Parameters	Odds	95% CI	*p* value	AIC
Model 2a (PQ-BC cutoff T1)	SDSC cutoff T0	1.621	1.41, 1.86	< 0.001	6548
Model 2b (PQ-BC cutoff T1)	SDSC cutoff T0	1.407	1.20, 1.65	< 0.001	6538
	SDSC cutoff T1	1.325	1.13, 1.56	0.001	
Model 2c (PQ-BC cutoff T1)	SDSC cutoff T0	1.496	1.29, 1.74	< 0.001	6048
	Gender_(RV=Male)_	0.799	0.70, 0.91	0.001	
	Ethnicity_(RV=White)_	1.031	0.98, 1.09	0.257	
	Socioeconomic status	1.171	1.09, 1.26	< 0.001	
	Neighbourhood deprivation	1.008	1.00, 1.01	< 0.001	
	IQ	0.959	0.94, 0.98	< 0.001	
	Family conflict	0.986	0.95, 1.02	0.430	
	Stimulant medication	1.649	1.26, 2.16	< 0.001	
Model 2d (PQ-BC cutoff T1)	SDSC cutoff T0	1.320	1.11, 1.57	0.001	6041
	SDSC cutoff T1	1.294	1.09, 1.53	0.003	
	Gender_(RV=Male)_	0.794	0.70, 0.91	0.001	
	Ethnicity_(RV=White)_	1.030	0.98, 1.09	0.267	
	Socioeconomic status	1.163	1.08, 1.25	< 0.001	
	Neighbourhood deprivation	1.008	1.00, 1.01	< 0.001	
	IQ	0.959	0.94, 0.98	< 0.001	
	Family conflict	0.983	0.95, 1.02	0.356	
Stimulant medication		1.605	1.22, 2.10	< 0.001	

*SDSC* Sleep Disorder Scale for Children, *PQ-BC* Prodromal Questionnaire-Brief Child version, *CI* confidence intervals, *AIC* Akaike information criterion

### RQ3: Do sleep disorders predict persistence of psychotic experiences?

The results of the logistic regression analyses relating to persistence of psychotic experiences are presented in Table 4. Presence of sleep disorder symptoms at baseline (OR 1.71, 95% CI 1.48–1.97) and persistence of sleep disorder symptoms (OR 1.89, 95% CI 1.60–2.23) were highly significant predictors of persistence of psychotic experiences at 12 months. These effects remained strong even once control variables were added (ORs of 1.56 and 1.72 for baseline and persisting sleep disorder symptoms, respectively). The pattern of results was similar when presence of distressing psychotic experiences was used as the outcome variable (unadjusted OR 1.81, 95% CI 1.51–2.17, adjusted OR 1.62, 95% CI 1.34–1.97 for persistence of sleep disorder symptoms).

**Table 4:** Regression analyses of whether sleep disorders at baseline predict persistence of psychotic experiences at 12 months

Model (outcome)	Parameters	Odds	95% CI	*p* value	AIC
Model 3a (PQ-BC persist)	SDSC cutoff T0	1.707	1.48, 1.97	< 0.001	6192
Model 3b (PQ-BC persist)	SDSC persist	1.891	1.60, 2.23	< 0.001	6188
Model 3c (PQ-BC persist)	SDSC cutoff T0	1.562	1.34, 1.82	< 0.001	5710
	Gender_(RV=Male)_	0.798	0.70, 0.91	0.001	
	Ethnicity_(RV=White)_	0.999	0.95, 1.05	0.957	
	Socioeconomic status	1.163	1.08, 1.25	< 0.001	
	Neighbourhood deprivation	1.009	1.01, 1.01	< 0.001	
	IQ	0.953	0.93, 0.98	< 0.001	
	Family conflict	0.989	0.95, 1.03	0.553	
	Stimulant medication	1.471	1.13, 1.92	0.005	
Model 3d (PQ-BC persist)	SDSC persist	1.716	1.44, 2.04	< 0.001	5706
	Gender_(RV=Male)_	0.796	0.70, 0.91	0.001	
	Ethnicity_(RV=White)_	0.998	0.95, 1.05	0.943	
	Socioeconomic status	1.160	1.08, 1.24	< 0.001	
	Neighbourhood deprivation	1.009	1.01, 1.01	< 0.001	
	IQ	0.952	0.93, 0.98	< 0.001	
	Family conflict	0.991	0.96, 1.03	0.627	
	Stimulant medication	1.460	1.12, 1.91	0.005	

*persist* above cutoff at T0 and T1, *SDSC* Sleep Disorder Scale for Children, *PQ-BC* Prodromal Questionnaire-Brief Child version, *CI* confidence intervals, *AIC* Akaike information criterion

### RQ4: Do sleep disorders predict remission of psychotic experiences?

Remission of sleep disorder symptoms was not a significant predictor of remission of psychotic experiences (OR 1.041, *p* = 0.766, 95% CI 0.80, 1.35) in the uncontrolled model, therefore the analysis with control variables was not carried out. Again, results were similar when predicting distressing psychotic experiences as an outcome (OR 1.06, *p* = 0.408, 95% CI 0.84–1.33, AIC 5123).

### Differences arising from weighted analysis

The results using the population weighted values are reported in Supplementary Material 3. The pattern of all results remained substantially the same except for non-white ethnicity which became a significant predictor of onset of psychotic experiences at 12 months.

## Discussion

The current study found that sleep disorder symptoms were strong and significant predictors of both the co-occurrence and persistence of psychotic experiences in 9- to 11-year-old, with associations remaining robust after controlling for potential confounders such as socioeconomic status, ethnicity, IQ, and stimulant medication. The prediction of persistence of psychotic experiences is especially relevant, given that the persistence of psychotic experiences through adolescence is associated with most severe mental health outcomes [42]. The remission of sleep disorders did not predict remission of psychotic experiences, and possible reasons for a lack of association are discussed below. The pattern of results remained consistent when only distressing psychotic experiences were included, and when weighting was applied. Overall the results provide further support for a relationship between sleep problems and psychotic experiences during preadolescence.

Psychotic experiences were common in our study—reported by almost two-thirds of participants at baseline. This is similar to previous studies in this age group [9], and the relatively high rate of remission versus new onset by the 1-year follow-up is also in keeping with existing developmental research of psychotic experiences [27, 43]. Sleep disorder symptoms were also relatively common in the study group, with just under a third of the cohort having significant sleep issues, similar to the 30–40% reported in previous studies [44]. Guidelines on child sleep difficulties tend to advise that they ameliorate over time, contrary to our findings that around two-thirds do not (at least over a 1-year observation period). There is evidence of increasing rates of child and adolescent sleep disorders over time [45] suggesting a need for additional support for parents and health services to address sleep difficulties.

The current study extends the known relationship between sleep and psychotic experiences to early adolescence and addressed potential confounders, yet mediating mechanisms require further investigation. A particularly relevant mechanism linking sleep and psychotic experiences is negative affect, which is typically found to either partially or totally mediate this relationship in adult studies [3, 6, 46, 47]. This and other potential mediators (such as affect dysregulation) should be investigated once further ABCD cohorts are available (or in other appropriate longitudinal designs), or ideally in manipulation studies to allow tests of the direction of influences over time in this age group.

The findings with respect to persistence of psychotic experiences are particularly relevant to clinical outcomes. As discussed above, the persistence of psychotic experiences across adolescence is associated with particularly poor outcomes [42, 48]. In addition to potential mediating mechanistic factors (not examined in this study) it seems highly plausible that sleep problems and psychotic experiences could interact and exacerbate each other—for example, distressing psychotic experiences may lead to disturbed sleep, which then lead to higher vulnerability for psychotic experiences the next day, as indicated by experience sampling studies in adults with psychotic disorders [49]. This potential causal model of sleep in contributing to psychotic experiences would support interventions on sleep to prevent persistence of psychotic experiences. These have been piloted in adolescents at-risk of psychosis, with initial indications that sleep treatment does improve psychotic experiences—but this is awaiting further examination in a follow-up trial currently underway [50, 51].

However, this current analysis did not support a relationship between the remission of sleep disorders and the remission of psychotic experiences. As this study was observational, it is not possible to confirm a (lack of) causal relationship although several hypotheses can be generated. The first is that sleep disorders may cause increase and persistence in psychotic symptoms through altering a mediating mechanism that remains when sleep disorders resolve. For example, affective dysregulation has been highlighted as a potential mediating factor [52] with some evidence that this may also be apparent in children [18], as was also highlighted in Paper 1. Alternatively, sleep disorders may cause sensitisation of the mechanisms that generate psychotic symptoms but more rapidly than desensitisation occurs when sleep disorders resolve, and although recovery may occur, it may need more than the year interval measured with this data. Mechanisms here may be neurocognitive (for example, arousal-based increases in aberrant salience) or social (for example, sleep disorder causing behaviour difficulties, exclusion, or victimisation, subsequently impacting on psychotic experiences). Nevertheless, this does raise uncertainty about the potential efficacy of sleep improvement intervention in reducing psychotic symptoms in preadolescent children, given than an association between improved sleep and reduced psychotic experiences would be expected in this study if there were a direct causal association.

Besides our primary focus on the influence of sleep on psychotic experiences, our findings support the association of a wide range of epidemiological factors (male gender, socioeconomic status, neighbourhood deprivation) with psychotic experiences in this age group. Lower socioeconomic status of parents and increased neighbourhood deprivation were consistent predictors as supported by previous research [53]. Non-white ethnicity was not found to be a significant predictor in the unweighted models but was significant in some analyses once the models were weighted, supporting the longstanding association reported elsewhere [33]. The results therefore support broader scale social interventions in relation to prevention in mental health, and further specific investigation of the mechanisms between, for example, experiences of deprivation or discrimination and the development of psychotic experiences. Lastly, the strong relationship between stimulant medication and psychotic experiences is in keeping with previous literature [54].

### Limitations

We note that the observational nature of this study limits the causal conclusions we can draw as we cannot rule out that any changes or associations observed are due to unobserved factors. The current observation is also limited only to 1 year, which may be considered a short period of time to observe longitudinal associations between exposure and outcome—raising the possibility that the 1-year associations are merely reflecting the concurrent links at baseline. In response to this we would point to the changes in sleep disorder and psychotic symptom occurrence over the study period as indicating change does occur in this time period, and therefore it is relevant to investigate the predictors of this variability.

We have been limited in variables available by the measures used in the ABCD study, which have their own benefits and limitations. For example, while self-reported scales for psychotic experiences in adolescence are recommended, false positives are known to occur [55, 56] and therefore these associations need to be additionally explored with alternative measures. The sleep measure was parent-reported and parents may vary in their detection of sleep disorders in their offspring, and some sleep disorders may be more likely to be observed by parents (e.g. sleep-walking versus insomnia).

In conclusion, this study indicates that sleep disorders in late childhood/early adolescence are common, and are associated with increased likelihood of reporting psychotic experiences both at the time and at 1-year follow-up. Sleep disorders were also strongly associated with persistence of psychotic experiences from baseline to 1 year follow-up—this is of particular importance given that persistence of psychotic experiences in this period is associated with later psychotic disorder. These associations remained even while controlling for a broad range of confounding variables. This study suggests further attention towards sleep disorders in this period both as a plausible contributing factor to later mental health problems, but also as a possible preventative treatment target.

## Supplementary Information

Below is the link to the electronic supplementary material.

Supplementary file1 (DOCX 23 KB)

Supplementary file2 (DOCX 32 KB)

Supplementary file3 (DOCX 52 KB)
